# Unveiling Atypical and Uncommon Appendiceal Pathologies in Pediatric Patients With Appendicitis

**DOI:** 10.7759/cureus.109804

**Published:** 2026-05-28

**Authors:** Suganya Nadarajan, Aftab Ahmed

**Affiliations:** 1 Pediatrics, Mediclinic Parkview Hospital, Dubai, ARE; 2 Pediatric Surgery, Mediclinic Welcare Hospital, Dubai, ARE

**Keywords:** appendiceal diverticulosis, appendiceal lipoma, appendicular actinomycosis, atypical appendicitis, uncommon presentation

## Abstract

Acute appendicitis typically presents with classic symptoms; atypical manifestations lacking distinct features are common. Failure to recognize vague presentations delays diagnosis and increases complications. We present a pediatric case series of three male patients aged seven, 12, and 15 years with confirmed appendicitis but unusual additional pathological findings only discovered after appendectomy. The seven-year-old patient exhibited severe diffuse tenderness with evidence of perforation, abscess, and marked inflammation; resection revealed multiple obstructing appendiceal lipomas underlying this complicated disease course. The 12-year-old patient had subtle symptoms, but operative findings showed significant localized inflammation; pathology identified predisposing appendiceal diverticulae. Finally, the 15-year-old patient had inflammation with *Actinomyces* colonization, representing an incidental discovery unrelated to infection in the setting of appendicitis. These cases illustrate that unusual pathological findings can sometimes underly or be incidentally associated with typical appendiceal inflammation. Thorough pathologic assessment facilitates clinical management when atypical features manifest with appendicitis. Improved provider recognition of the heterogeneity of appendicitis presentations enables timely diagnosis even absent classic features.

## Introduction

Appendicitis, defined as inflammation of the vermiform appendix, is a very common surgical emergency with a lifetime disease risk of approximately 7%. It is one of the most prevalent gastrointestinal conditions necessitating urgent operation [1]. Typically, patients present with peri-umbilical or epigastric abdominal pain that later shifts to and localizes in the right lower quadrant (RLQ) [2]. The classic presentation is accompanied by anorexia, nausea, and vomiting. Migratory pain localizing to the RLQ and focal peritoneal signs on exam indicate likely appendiceal inflammation. This classic presentation facilitates rapid diagnosis. However, atypical presentations lacking these features are common, occurring in up to 30% of cases. Failure to promptly recognize atypical appendicitis delays diagnosis and increases the risk of perforation [3]. 

When appendicitis is not definitively diagnosed on initial assessment, observation, and serial abdominal exams helps reassess disease evolution in 12-24 hours. During this observation period, advanced imaging, such as a computed tomography (CT) scan, can also aid in diagnosis [4]. Once appendicitis is strongly suspected, surgical removal is indicated even before perforation or abscess development, given the rapidity of appendiceal gangrene. Urgent operation significantly improves patient outcomes and reduces complications like perforation or sepsis [1]. However, increased time to diagnosis negatively impacts morbidity with increased hospital length of stay, higher infection rates, and poorer recovery after surgery [4]. Thus, it is critical for providers in all specialties assessing patients presenting with abdominal pain to maintain a strong clinical suspicion for atypical appendicitis.

In this case series, we present patients exhibiting non-classical appendicitis manifestations seen at our hospital over the past year. For each case, we discuss key aspects of history, physical exam, and workup to highlight clinical pearls facilitating timely diagnosis even in the absence of classic features. Improved physician familiarity with the heterogeneous manifestations of appendicitis is pivotal to enable accurate diagnosis and prevent perforation-related complications, even when patients do not present with textbook symptoms. 

## Case presentation

Case 1

A seven-year-old male patient presented with a fever for one day, accompanied by vomiting and severe suprapubic abdominal pain. The dysuria pain score was noted as 10/10. A day prior, a urinalysis was performed, showing normal results. On examination, his abdomen was diffusely tender, especially in the RLQ and suprapubic regions, with involuntary guarding and positive rebound tenderness.

**Figure 1:**
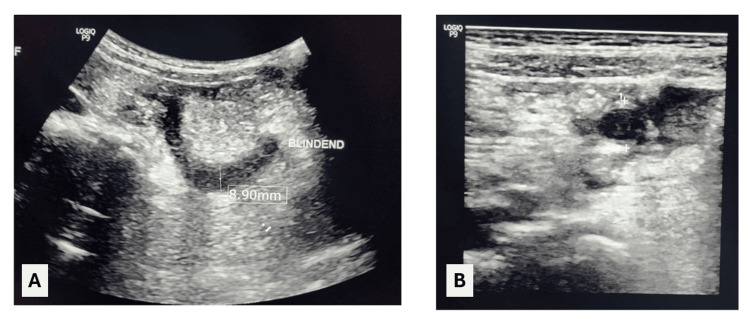
Ultrasonography images suggestive of appendicitis with free fluid

Initial labs were significant for leukocytosis of 20 (normal range 5-13 10^3^/ uL) and an elevated C-reactive protein (CRP) of 137 mg/L (normal range 0-5 mg/L). An abdominal ultrasound showed a thickened appendix concerning appendicitis, reactive mesenteric lymphadenitis, and a mild free fluid in the right iliac fossa (Figure 1).

**Figure 2:**
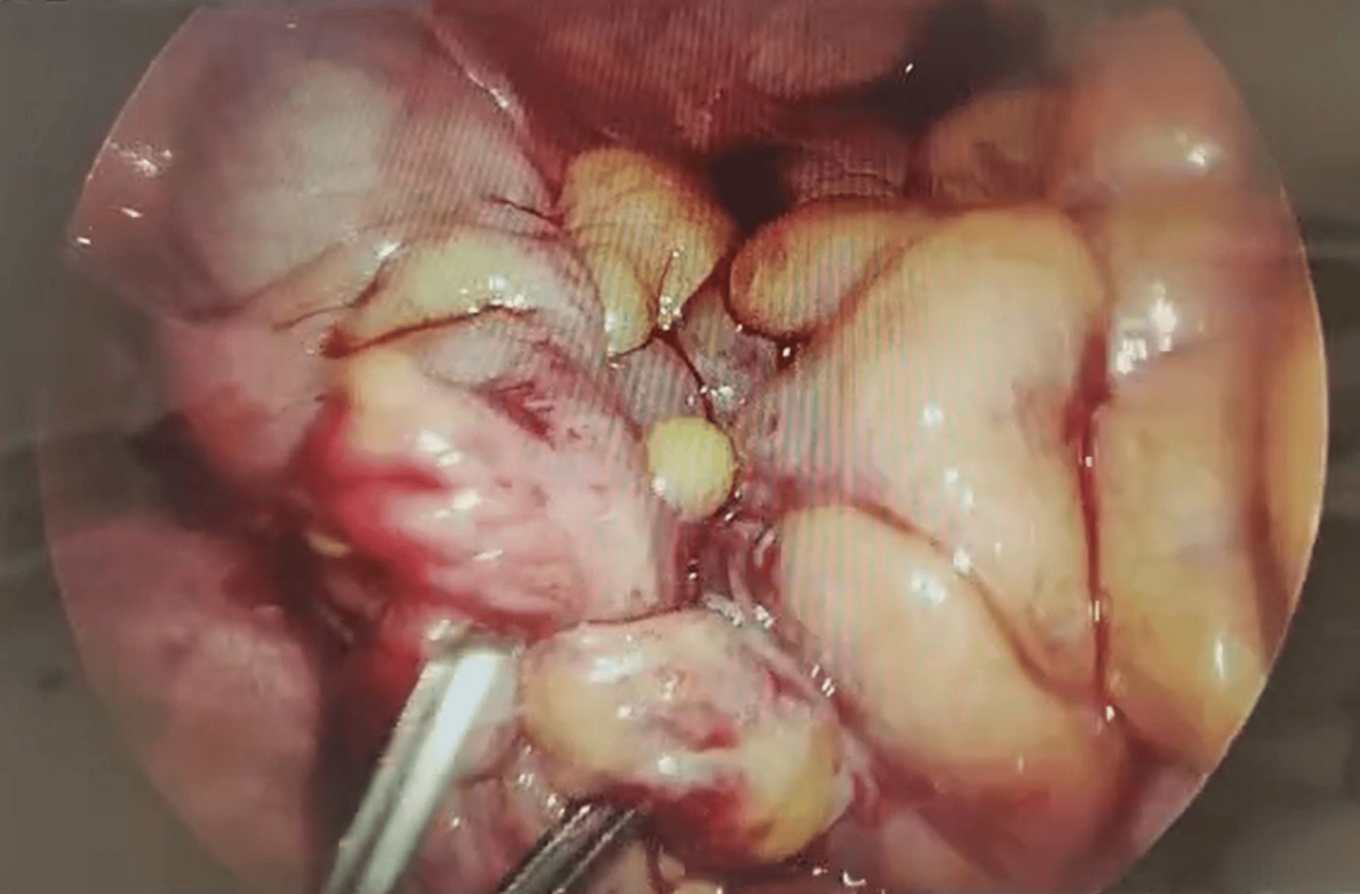
Laparoscopic finding of lipomas

The patient underwent laparoscopic appendectomy with lipoma removal (Figure 2). The findings during surgery led to a provisional diagnosis of acute appendicitis with generalized peritonitis and multiple lipomas. The histopathology report indicated that the appendiceal tissue had a heavy transmural neutrophilic infiltrate, extending into the subserosa and mesoappendix. Abundant mature fibro-adipose tissue was observed in association with the appendix. However, there were no morphologic features indicating neuroendocrine proliferation, dysplasia, or malignancy.

Case 2

A 12-year-old boy presented with one day of abdominal pain and some loose stools over the past 12 hours. On examination, he had right iliac fossa tenderness. Labs were significant for a white count of 19 (normal range 4-11 10^3^/ uL) and CRP of 81 mg/L (normal range 0-5 mg/L).

**Figure 3:**
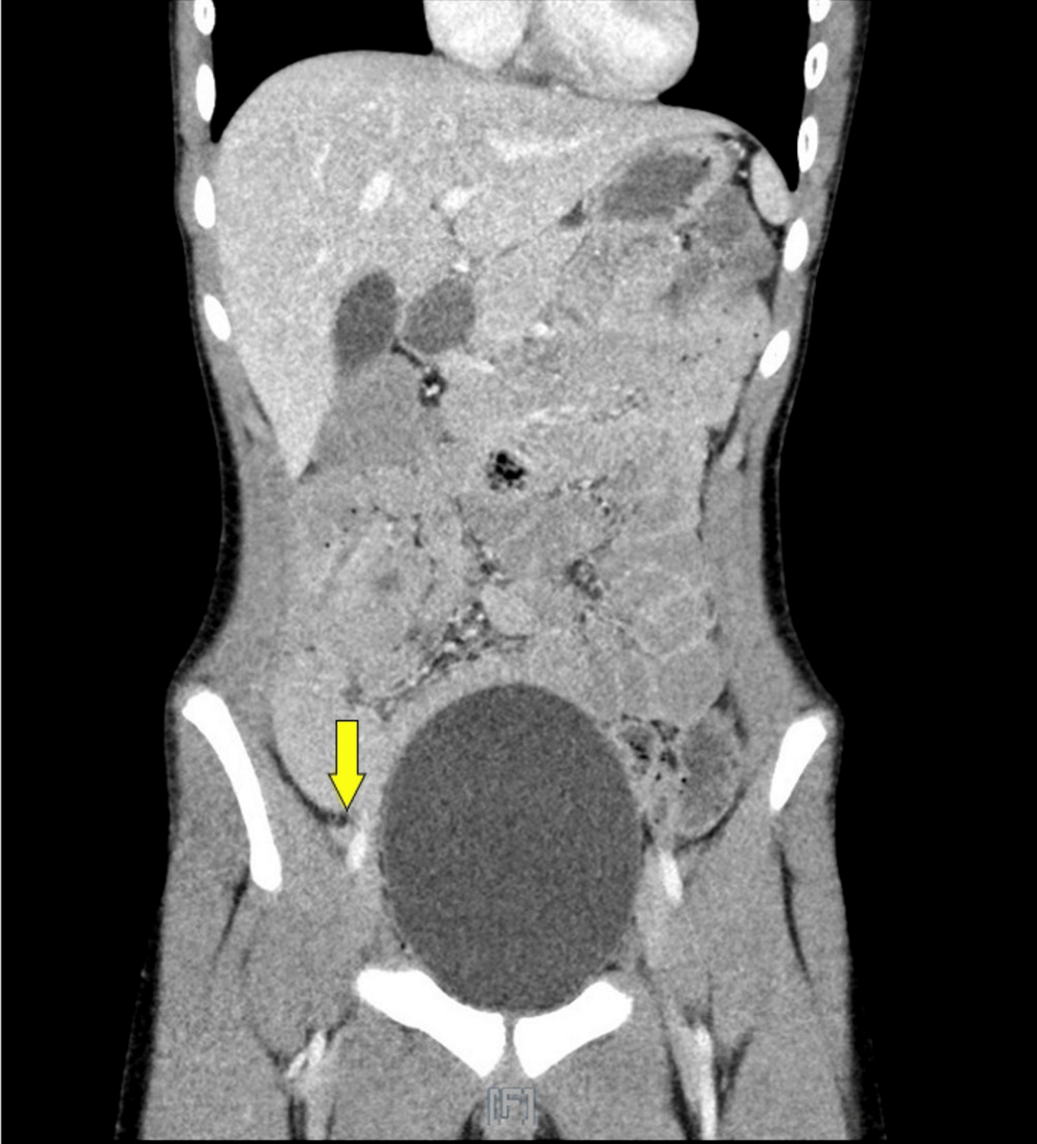
CT abdomen showing retrocecal appendicitis along diverticulae

During the abdominal ultrasound, both kidneys showed slight echogenicity, and there was mild hydronephrosis in the left kidney. The appendix was not visualized, but tenderness in the right iliac fossa and a small amount of fluid were observed. The CT abdomen with contrast showed retrocecal appendicitis with minimal fat stranding (Figure 3).

**Figure 4:**
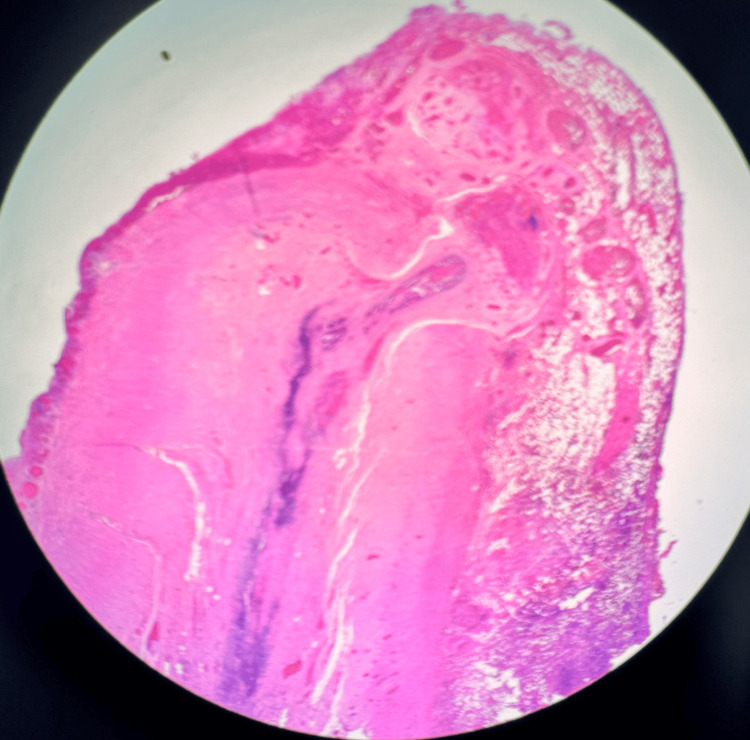
Histopathology image of the appendiceal diverticulum

The patient underwent a laparoscopic appendectomy. The histopathology report revealed acute suppurative appendicitis and periappendicitis with focal hemorrhage. Additionally, there were features indicating appendiceal diverticulae (Figure 4), and no malignancy was detected.

Case 3

A 15-year-old previously healthy male presented with two days of constant right lower abdominal pain associated with vomiting.

**Figure 5:**
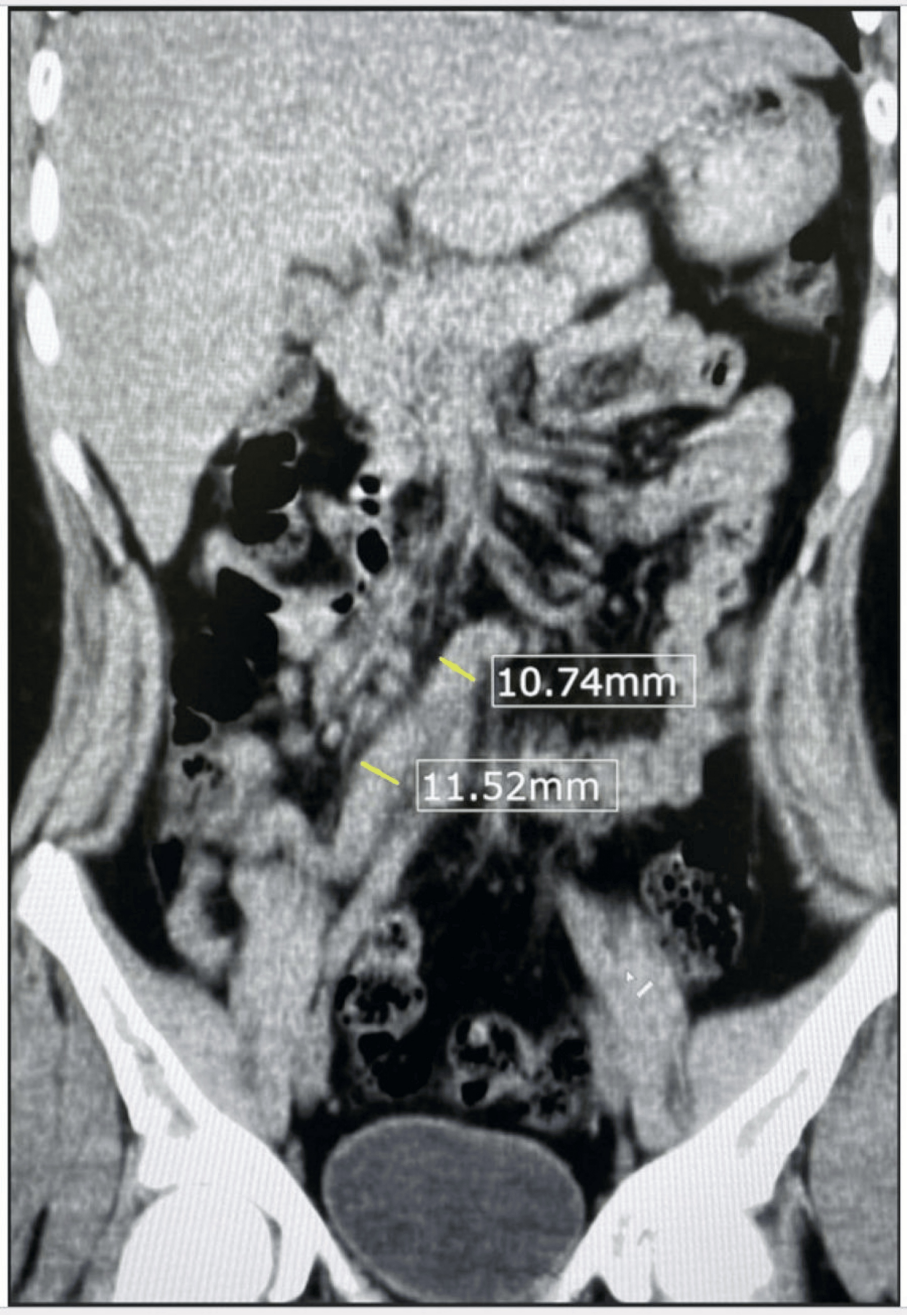
CT Abdomen findings consistent with acute appendicitis

**Figure 6:**
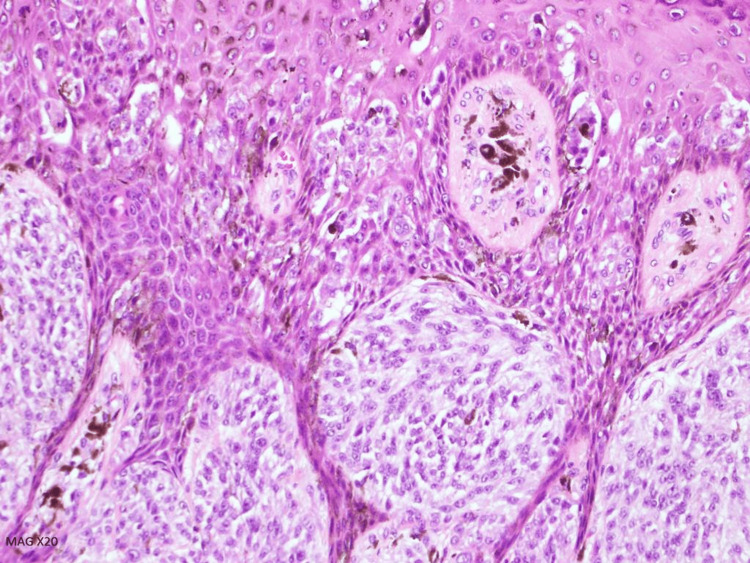
Histopathology image showing Actinomyces

On examination, he was found to have right iliac fossa tenderness. Labs showed white cell count of 13 (normal range 4-11 10^3^/ uL) and CRP of 60 (normal range 0-5 mg/L). A CT abdomen demonstrated signs of appendiceal inflammation consistent with acute appendicitis (Figure 5). The patient subsequently underwent laparoscopic appendectomy and peritoneal lavage. The histopathology report indicated the presence of *Actinomyces* in the appendix tissue (Figure 6). No malignancy was detected.

## Discussion

Acute appendicitis is one of the most common surgical emergencies in children. Though typically involving obstruction and inflammation, unusual pathological findings can sometimes be elucidated after appendiceal resection. This report presented three cases of confirmed appendicitis in pediatric male patients with unique additional pathology, including appendiceal lipomas, diverticulae, and *Actinomyces* organisms.

Appendiceal lipomas are extremely rare and consist of abnormal proliferation and accumulation of mature adipocytes attached to the appendix [5]. Though usually incidental, appendiceal lipomas can contribute to obstruction and inflammation. This was likely the cause in Case 1, where the patient presented with severe, diffuse abdominal pain and acute perforated appendicitis with concerning exam findings, marked leukocytosis, and substantial imaging evidence of inflammation. His operative findings were consistent with advanced disease, and, notably, the gross specimen demonstrated multiple small lipomas necessitating additional resection. Though an unusual finding, this elucidates how obstruction from lipomas can lead to complicated appendicitis.

Appendiceal diverticulae are likewise uncommon, with a 0.004% incidence rate [6]. They involve herniation and protrusion of the appendiceal mucosa through defects in the muscularis propria. Diverticula can predispose to obstruction, stasis, and inflammation [7]. In Case 2, the patient presented with migratory pain and evidence of early inflammation both on CT and ultimately during surgery. While pathology showed acute suppurative appendicitis, there were multiple diverticulae emanating from his appendix. These could explain his symptoms and serve as a risk factor for potential recurrent issues in the future.

Finally, *Actinomyces* is a genus of anaerobic gram-positive bacteria that normally reside in the gastrointestinal tract. Actinomycosis causes granulomatous lesions with associated abscesses and fibrosis [8]. Although *Actinomyces *predominantly cause cervicofacial disease, they can also rarely cause intra-abdominal infection. However, in Case 3, the patient had simple appendiceal inflammation without signs of associated infection. Therefore, the *Actinomyces* found incidentally within his appendix likely represented benign colonization rather than true pathology. Nevertheless, due to his recent inflammation, antibiotics were administered to prevent issues should perforation or leakage later occur.

Overall, while appendicitis itself is common, these cases illustrate that atypical findings can be occasionally encountered. Associated lipomas, diverticulosis, or organism growth may contribute mechanistically to obstruction and inflammation. Alternatively, they may simply represent incidental discoveries in already inflamed appendiceal tissue. However, identifying these associations is valuable both for directing management and for better appreciating the scope of appendiceal disease presentations, especially in the pediatric population. Certain findings may necessitate additional resection, antibiotic administration, or heightened surveillance to prevent recurrence. Above all, these cases highlight the utility of routine surgical pathology analysis, even in presumed simple appendicitis. Additional clinical-pathologic correlation and discussion at interdisciplinary conferences could further enhance patient care and expand medical education on these rare manifestations.

## Conclusions

We presented three pediatric appendicitis cases with unusual pathological findings; the first associated with multiple obstructive lipomas, the second with predisposing diverticulae development, and the third with colonizing *Actinomyces* of unclear significance in the setting of inflammation. Pathologic assessment proved invaluable in guiding management for optimal outcomes across all cases. Greater provider awareness of such atypical findings can better standardize future care for these rare appendicitis subtypes.
